# Myosin IXB protects immune cells from virus infection

**DOI:** 10.1099/jgv.0.002090

**Published:** 2025-04-01

**Authors:** Leticia Kogachi, Taís Matozo, Yuli Thamires Magalhães, Marina Janoni Bayerlein, Tania Carolina Braga, Felippe E. C. Camargo, Kamilla B. da S. Souza, Fábio Luis Forti, Bruna Cunha de Alencar

**Affiliations:** 1 Laboratory of Cell Biology of the Immune System, Departamento de Imunologia, Instituto de Ciências Biomédicas, Universidade de São Paulo, São Paulo, Brazil; 2 Laboratory of Signaling in Biomolecular Systems, Departamento de Bioquímica, Instituto de Química, Universidade de São Paulo, São Paulo, Brazil

**Keywords:** actin, cytoskeleton, infection, myosin 9B, RhoGTPases, virus

## Abstract

Actin-associated proteins have been implicated in several stages of virus infection. However, the role of myosins, which are actin-dependent molecular motors, during virus infection and pathogenesis is poorly understood. Myosin IXB (Myo9b) is a member of the myosin family abundantly expressed in immune cells. Myo9b displays a RhoGTPase-activating protein domain capable of modulating actin dynamics by inhibiting RhoGTPase activity. To enquire upon Myo9b participation in virus infections, we have silenced Myo9b in U937 and Jurkat cells and infected them with vesicular stomatitis virus glycoprotein (VSV-G)-pseudotyped HIV-1. Myo9b-silenced U937 showed a remarkable increase of above ten times more HIV-VSV-G infection than control cells. We observed a similar pattern in Jurkat cell infection with both WT Env and VSV-G-pseudotyped HIV, albeit to a lesser extent. Myo9b-silenced U937 cells presented elevated levels of phosphorylated cofilin, but lower levels of polymerized actin. The use of a RhoA, B and C inhibitor, as well as a Rac1 inhibitor, reduced virus infection. Finally, we have also observed an increment in virus internalization and fusion in cells knockdown for Myo9b, which may explain the increase in virus infection. Taken together, our data suggests that Myo9b might hinder viral entry and infection by controlling the activity of RhoGTPases in immune cells.

## Introduction

Several pieces of evidence have shown that regulation of the actin cytoskeleton is key for the successful infection of cells by viruses, including the human immunodeficiency virus type 1 (HIV-1) [1,2]. Myosins are actin-associated molecular motors capable of breaking ATP molecules and using the resulting energy to produce movement, which triggers their displacement on actin tracks or movement of actin filaments. Mammalian cells express several myosins that belong to different classes and have unique functions. Myosins are involved in cell division, vesicle transport, endo- and exocytosis, regulation of membrane tension and cell migration, amongst several others [3]⁠. Despite the clear involvement of actin in virus infections and the broad range of processes carried out by myosins, little is known about each myosin’s function in virus infections [4]⁠.

Myosin IXB (Myo9b) is one of two members of the myosin IX family in humans. Myosin IX motors are monomeric, and their most distinctive feature is the presence of a RhoGTPase-activating protein (RhoGAP) domain [5]⁠. Small RhoGTPases are a family of proteins that cycle between an active, guanosine triphosphate(GTP)-bound state and an inactive, guanosine diphosphate (GDP)-bound state. Their most prominent members include RhoA, Rac1 and Cdc42. RhoGTPases are molecular switches for several signalling pathways and are essential for the control of the actin cytoskeleton. RhoGAPs induce hydrolysis of the Rho-associated GTP molecule into GDP and thus inactivate RhoGTPases (reviewed by [6⁠]).

Myo9b is highly expressed by cells of the immune system, but also by intestinal epithelia, and is up-regulated in some tumours [7,12]⁠. It has been implicated in cell morphogenesis and also in the motility of osteoclasts, T lymphocytes and dendritic cells [13,17]⁠. Finally, polymorphisms in *MYO9B* have been associated with several inflammatory diseases, especially in the digestive system, including coeliac disease, inflammatory bowel disease, ulcerative colitis and Crohn’s disease [18,24]⁠.

The RhoGAP domain in Myo9b inhibits RhoA, RhoB and RhoC [25]⁠. Human Myo9b may also inhibit Rac1 and Cdc42 [26]⁠. Despite being monomeric, Myo9b can walk processively on actin filaments due to a second actin-binding domain on its head [27]⁠. Those characteristics result in a RhoGAP that is capable of displacement on actin tracks and therefore may be rapidly recruited to control RhoGTPase activation in discrete locations of the cell.

In this work, we used the monocytic cell line U937 infected by HIV-1 pseudotyped with vesicular stomatitis virus glycoprotein (VSV-G) as a model to enquire upon the role of Myo9b in viral infection. We found that the knockdown of Myo9b significantly increased the percentage of cells that became infected. Myo9b-silenced lymphocytic Jurkat cells were also more susceptible to infection by both HIV carrying WT Env and VSV-G-pseudotyped virus, although not to the same extent as U937 cells. U937 cells silenced for Myo9b displayed increased levels of phosphorylated cofilin (p-cofilin), suggesting more active RhoGTPases. On the other hand, Myo9b-silenced U937 showed weaker phalloidin staining of polymerized actin. These cells also presented higher virus fusion and internalization. We conclude that, in the absence of Myo9b, more active RhoGTPases led to increased virus internalization and subsequent infection. This study suggests that the control exerted by Myo9b over the RhoGTPase system can be important in protecting immune cells from virus infections.

## Methods

### Cell lines and cell culture

U937, Jurkat E6.1 and Ghost X4/R5 were obtained from the NIH AIDS Reagent Program. U937 and Jurkat cells were maintained in complete Roswell Park Memorial Institute medium (RPMI, Gibco), i.e. supplemented with 10% fetal bovine serum (FBS, Gibco), penicillin and streptomycin (Gibco). HEK293T, kindly provided by Dr. Enrique Mario Boccardo Pierulivo from ICB/USP, and Ghost X4/R5 were kept in complete DMEM GlutaMax (Gibco).

Primary cells were obtained from leucocyte reduction chambers that were recovered after platelet donations in the blood bank of Hospital Alemao Oswaldo Cruz. Donors consented to the use of their cells in the research project studying myosins.

### Plasmids

Plasmids pNL4-3 (REF 114), pNL deltaE EGFP (REF 11100), pVSV-G (REF 4693), pBlaM-Vpr (REF 11444), pGag-iGFP (REF 12457), psPAx2 (REF 11348) and pNL4-3 deltaE EGFP (REF 11100) were obtained from the NIH AIDS Reagent Program. pSIV3+ was generously provided by Dr. Nicolas Manel, whilst pNL4-3.IRES.GFP was a kind gift from Dr. Philippe Benaroch (both NM and PB are from Institut Curie, Paris, France). pGag-GFP was generously provided by Dr. Luis Lamberti Pinto Silva, from the University of Sao Paulo Medical School in Ribeirao Preto, Brazil. Mission short hairpin RNA (shRNA)-specific plasmids were obtained from Sigma-Aldrich. shRNA sequences are shown in Table 1.

**Table 1.** shRNA sequences

Name	Sigma ID	Short hairpin sequence
shSCRAMBLE	SHC002	CCGGCAACAAGATGAAGAGCACCAACTCGAGTTGGTGCTCTTCATCTTGTTGTTTTT
shMYO9B #1	TRCN0000007138	CCGGGCCCATTGAGAGCTTGTTTATCTCGAGATAAACAAGCTCTCAATGGGCTTTTT
shMYO9B #2	TRCN0000007141	CCGGGCACGTCAAGTTCCAGAACAACTCGAGTTGTTCTGGAACTTGACGTGCTTTTT
shMYO9B #3	TRCN0000273362	CCGGGCCCATTGAGAGCTTGTTTATCTCGAGATAAACAAGCTCTCAATGGGCTTTTTG
shMYO9B #4	TRCN0000273359	CCGGACGTGGTTTACGTAACTTTAACTCGAGTTAAAGTTACGTAAACCACGTTTTTTG
shMYO9B #5	TRCN0000273294	CCGGGCACGTCAAGTTCCAGAACAACTCGAGTTGTTCTGGAACTTGACGTGCTTTTTG

### Virus and lentiviral vectors

Viruses were produced by transfecting HEK293T with pNL4-3, pNL4-3.IRES.GFP or pNL4-3-EGFP (and pVSV-G for pseudotyped viruses). For fusion experiments, pBlaM-Vpr was also co-transfected with the chosen virus plasmid. For the production of lentiviral vectors for shRNA silencing, HEK293T cells were transfected with pshRNA, psPAx2 and pVSV-G. For viral-like particle (VLP) production, psPAX2, pGag-GFP and pVSV-G were co-transfected in HEK 293 T cells, using five times the molar amount of psPAX2 than pGag-GFP in order to get well-formed, fluorescent VLPs. All transfections were carried out using polyethylenimine (PEI, 25 kDa, linear, Polysciences Inc.). Briefly, plasmid DNA and PEI were diluted in NaCl 150 mM in separate tubes (the amount of PEI was 2.6× the mass of DNA). They were then mixed 1 : 1 and incubated for 20 min, before being added to HEK293T cells. Sixteen hours later, HEK293T medium was exchanged, and cells were further incubated for 24 h. The supernatant was collected, filtered through 0.45 µm filters and centrifuged at 10,000 ***g*** for 2 h at 4 °C overlaid on a Tris, NaCl and EDTA buffer containing 20% sucrose. The virus was titrated using increasing volumes to infect the reporter cell line Ghost X4/R5 cells, which were assessed 24 h later for GFP expression by flow cytometry.

VLPs were quantified using a p24 cytometric bead assay made in-house, as described by [28]⁠.

### Chemical compounds and antibodies

Phalloidin labelled with AF647 or rhodamine was acquired from Invitrogen.

Anti-Myo9b was acquired from Proteintech (12432-1-AP). Anti-p-cofilin was from Santa Cruz Biotechnology (sc-271921). Anti-*β*-tubulin and glyceraldehyde-3-phosphate dehydrogenase (GAPDH) were from Abcam (AB6046 and AB9484). Anti-low-density lipoprotein receptor(LDLR) was from Santa Cruz Biotechnology (SC-18823 AF 647). Anti-HIV p24 was from Beckman Coulter (KC57 FITC or Rd-1).

The membrane-permeable C3 inhibitor was obtained from Cytoskeleton Inc. (CT04), whilst NSC23766 was obtained from Santa Cruz Biotechnology.

### Cell transduction and infection

U937 and Jurkat E6.1 were transduced with lentivirus at least 2 days before selection with 1 µg ml^−1^ of puromycin. Transduced cells were then constantly kept in a medium containing puromycin.

For HIV infection, a multiplicity of infection (MOI) of 0.05 was used for U937 cells. For Jurkat E6.1, an MOI of 0.5 was used for NL4-3.IRES.GFP and of 0.1 for NL deltaE EGFP VSV-G. For fusion experiments, MOIs of between 0.01 and 0.05 were used. After infection, cells were kept for an additional 48 h unless stated otherwise. When viruses used for infection expressed GFP, cells were harvested, washed in phosphate-buffered saline (PBS), fixed in paraformaldehyde 1% and analysed by flow cytometry.

Part of the cells in each experiment was used for viability assays using the kit Cell Titer Glo (Promega). Briefly, the same volume of cells from each well was pipetted into a 96-well white plate, and 50 µl of Cell Titer Glo solution was added at room temperature for 30 min. The plate was then read at a luminometer, using only medium as a negative control.

For RhoA, B and C inhibition, membrane-permeable C3 was added at 2 µg ml^−1^ to U937 cells in serum-free RPMI and incubated for 4 h before infection by the addition of NL4-3 IRES GFP VSV-G at MOI 0.01. Cells were kept in a serum-free medium overnight, then washed once in PBS, resuspended in RPMI with 10% FBS and incubated for a further 24 h. The same procedure was carried out for Rac1 inhibition, except NSC23766 was added at 100 µM to U937 cells in RPMI with 2% FBS. In both cases, at 48 h post-infection, cells were then harvested, washed in PBS, stained with Live/Dead (Thermo Fisher Scientific), washed, fixed in paraformaldehyde 1% and analysed by flow cytometry.

### VLP internalization assay

For VLP internalization, cells were plated at 4 °C or 37 °C and incubated for 30 min to reach the desired temperature. Then, VLPs were added to the cells and incubated for 2 h. After this period, cells were washed in cold PBS twice. Some samples were then incubated with trypsin solution for 3 min at 37 °C; then, complete RPMI was added, and cells were centrifuged and washed in PBS. Finally, samples were fixed in 1% paraformaldehyde.

### Fusion assay

The fusion assay was performed as previously described [29]. In brief, cells were pulsed with NL4-3 BlaM-Vpr VSV-G for 2 h at 37 °C, washed twice with PBS and incubated with CCF4-AM solution for 1 h at room temperature. Cells were washed in a CO_2_-independent medium, resuspended in a developing solution (probenecid in CO_2_-independent medium) and incubated overnight at room temperature. Finally, cells were stained for viability, fixed and analysed by flow cytometry. For analysis, cells were gated in the FCS-A×SSC-A dot plot; then, the singlets were selected in the FSC-A×FSC-H plot, viable cells were gated and, finally, the cleaved substrates were gated in the Pacific Blue-A×FITC-A dot plot considering uninfected cells as the negative gate.

### Flow cytometry

Cells were read in a FACS Canto II or in an Accuri C6 (both from BD Biosciences). A first gate was drawn on the population defined by FSC and SSC. Then, we chose only single cells by analysing FSC-A×FSC-H. In fusion assays as well as in infection assays using RhoGTPase inhibitory drugs, cells were also stained with Live/Dead (Thermo Fisher Scientific), so a gate was made on live cells. The target fluorescence was then analysed.

### Quantitative PCR (qPCR)

RNA was extracted using NucleoSpin RNA (MACHEREY-NAGEL). One microgram of RNA was then reverse-transcribed to cDNA using the High Capacity cDNA Reverse Transcription Kit (Applied Biosystems), according to the manufacturer’s instructions. We then used 0.5 µl from the reverse transcription reaction to amplify the gene of interest using TaqMan primers/probes and TaqMan Universal Master Mix II (Applied Biosystems). The primers/probes used were purchased from Applied Biosystems and are described in Table 2.

**Table 2.** Taqman gene expression assays used for qPCR

Gene	TaqMan primer/probe
*Myo1c*	Hs00300761_m1
*Myo1e*	Hs00192232_m1
*Myo1f*	Hs00300741_m1
*Myo1g*	Hs01070818_m1
*Myh9*	Hs00159522_m1
*Myo5a*	Hs00165309_m1
*Myo7a*	Hs00934542_m1
*Myo9a*	Hs01003056_m1
*Myo9b*	Hs00188109_m1
*Myo10*	Hs01586613_m1
*Myo18a*	Hs00373018_m1
*B2M*	Hs00187842_m1

Samples were amplified using a QuantStudio 3 (Applied Biosystems) and the temperature cycle recommended by the mastermix manufacturer.

Gene expression was relative to the housekeeping gene beta-2-microglobulin (*B2M*) and calculated using the formula: 2^−(Ct gene of interest−Ct of housekeeping gene).

### Western blotting

To obtain cell lysates, cells were harvested, washed with PBS and incubated with 0.5% Igepal lysis buffer (50 mM Tris, pH 7.4 and 5 mM MgCl_2_) containing a protease inhibitor cocktail with EDTA (Pierce Protease Inhibitor Mini Tablets) for 30 min in ice. For p-cofilin, we also added a phosphatase inhibitor that was used according to the manufacturer’s instructions (PhosSTOP, Sigma). After protein extraction, protein content was quantified by Coomassie Blue Protein Assay Reagent (Thermo Scientific).

For the Myo9b Western blot (WB), polyacrylamide gels were 6.5% and contained 10% glycerol. After electrophoresis, proteins were transferred to a PVDF membrane (Thermo Scientific) by wet transfer for 2 h using transfer buffer (25 nM Tris, 192 nM glycine, 0.02% SDS and 10% methanol). For other WB, polyacrylamide gels were 10%.

The membrane was then blocked in Tris-buffered saline (TBS) with a 5% non-fat milk solution. Primary and secondary antibodies were diluted in the same blocking solution and incubated with the membrane for 1 h at room temperature each, with a 30 min wash in TBS-Tween 0.1% between antibody incubations. Anti-Myo9b was diluted 1 : 500, and anti-*β*-tubulin and anti-p-cofilin were diluted 1 : 1,000. Membranes were developed using ECL Clarity Max (Bio-Rad), and images were acquired in a LAS4000 (GE). Quantification was done using FIJI.

### Epifluorescence and confocal microscopy

To obtain microscopy images, cells were adhered to poly-d-lysine-treated coverslips (poly-d-lysine was purchased from Gibco). Briefly, the poly-d-lysine solution was diluted 1 : 1 in sterile PBS, and then 200 µl was added on the top of the coverslips to cover each completely. After 1 h incubation, coverslips were washed three times with sterile water and left to dry inside the hood. One hundred thousand cells were then resuspended in PBS and incubated on top of the poly-d-lysine-treated coverslips for 20 min and then fixed with 4% paraformaldehyde for 1 h. After fixation, cells were washed in PBS and permeabilized in a 0.2% BSA-0.05% saponin solution. Staining was performed first with a primary antibody diluted in BSA-saponin for 1 h at room temperature. After extensive washing with the BSA-saponin solution, cells were incubated with secondary antibodies diluted in the same solution for 1 h at room temperature, washed extensively and mounted on a DAPI-containing mounting medium (Vectashield, VectorLabs).

Wide-field microscopy images were acquired on a Zeiss AxioVert.A1 with an AxioCam lcm1, or on a Leica DMi8. Confocal images were acquired on a Zeiss LSM 800. Images were analysed using FIJI and LAS X Office.

For microscopy projections, images from the same field were obtained at different focal planes (0.4 µm between images in Z) using an epifluorescent microscope. The Z-stack was batch-blind deblurred and deconvoluted, and then processed into a single overlaid picture using the LAS X Office software algorithm. Each Z-stack had between 32 and 34 layers.

### Statistical analysis

For statistical analyses, we used the software GraphPad Prism. The appropriate statistical analysis conducted for each experiment is mentioned in each figure legend.

## Results

### Myosins’ expression in the monocytic cell line U937

In order to characterize the role of myosins in virus infections in myeloid cells, we chose the monocytic cell line U937 as a model. U937 can be differentiated into macrophages (a relevant HIV target cell) and even present some characteristics of HIV-infected macrophages, such as virus-containing compartments [30,31]⁠.

**Fig. 1.**
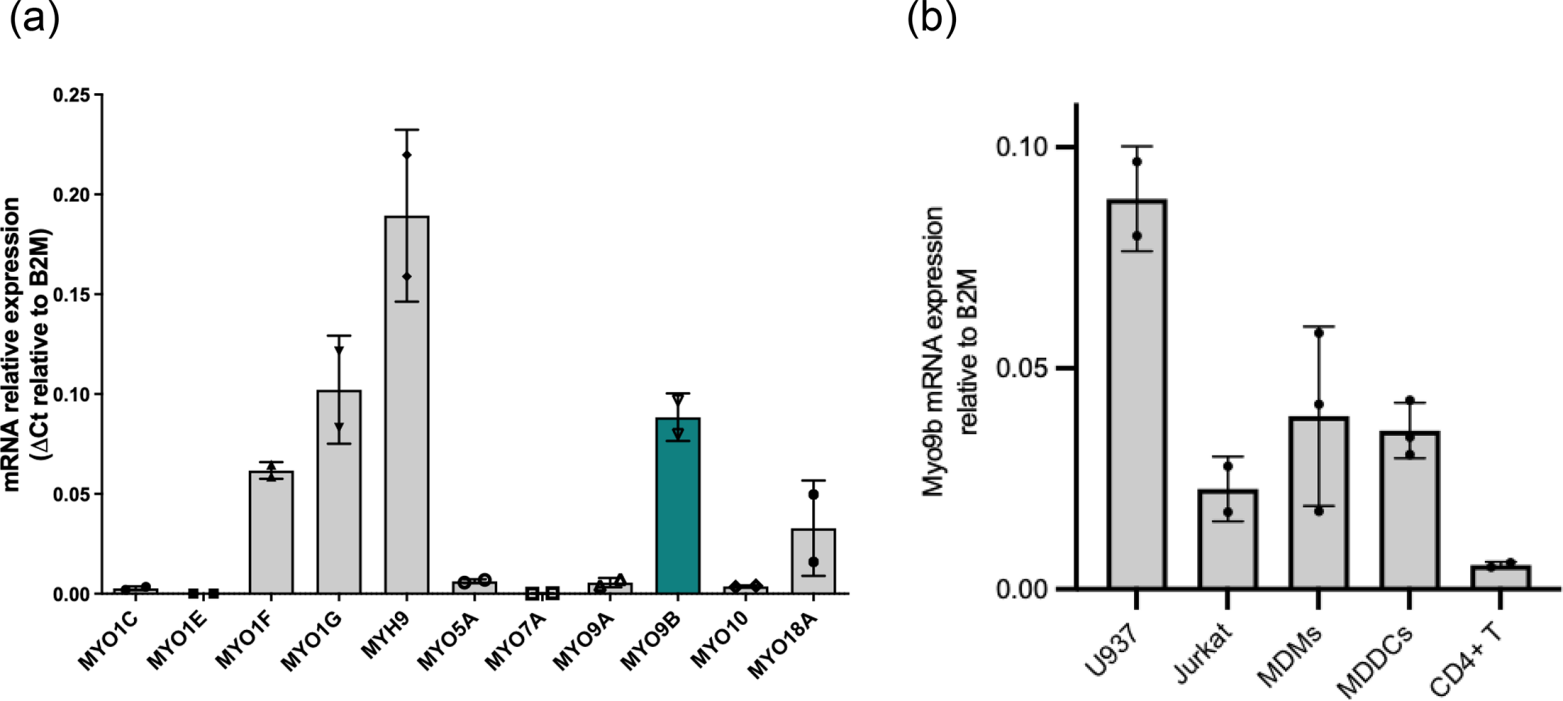
Myo9b expression by qPCR using TaqMan probes. (a) Expression of 11 myosins’ mRNA in U937 cells. The graph depicts the mean and sd for two independent experiments. (b) Expression of Myo9b RNA in different cell types/cell lines. The graph depicts the mean and sd for two (U937, Jurkats and CD4+ T lymphocytes) or three [monocyte-derived macrophages (MDMs) and monocyte-derived dendritic cells (MDDCs)] independent experiments. Relative expression (RE) was calculated using the formula RE=2^−(Ct gene of interest−Ct of B2M).

We started by characterizing the expression of different myosins in U937. We have tested the expression of 11 myosins that had been reported in murine hematopoietic cells [32]⁠. Of the 11 myosins tested, nine had their mRNA amplified, five of which at considerable levels (Myo1f, Myo1g, MYH9, Myo9b and Myo18a, Fig. 1a). We noticed that Myo9b was among the myosins expressed at higher levels in U937. We then compared Myo9b expression in cell lines used for the study of HIV infection, as well as in primary cells that are targets for the virus. As seen in Fig. 1(b), expression in U937 was highest. Monocyte-derived macrophages and dendritic cells expressed ~44% and 41% (respectively) of the levels seen in U937, whilst Jurkat cells, a CD4+ T cell line, expressed ~25% of the levels detected in U937. The expression was lowest in primary CD4 T cells.

### Myo9b knockdown resulted in a higher percentage of U937 cells infected by HIV-VSV-G

**Fig. 2.**
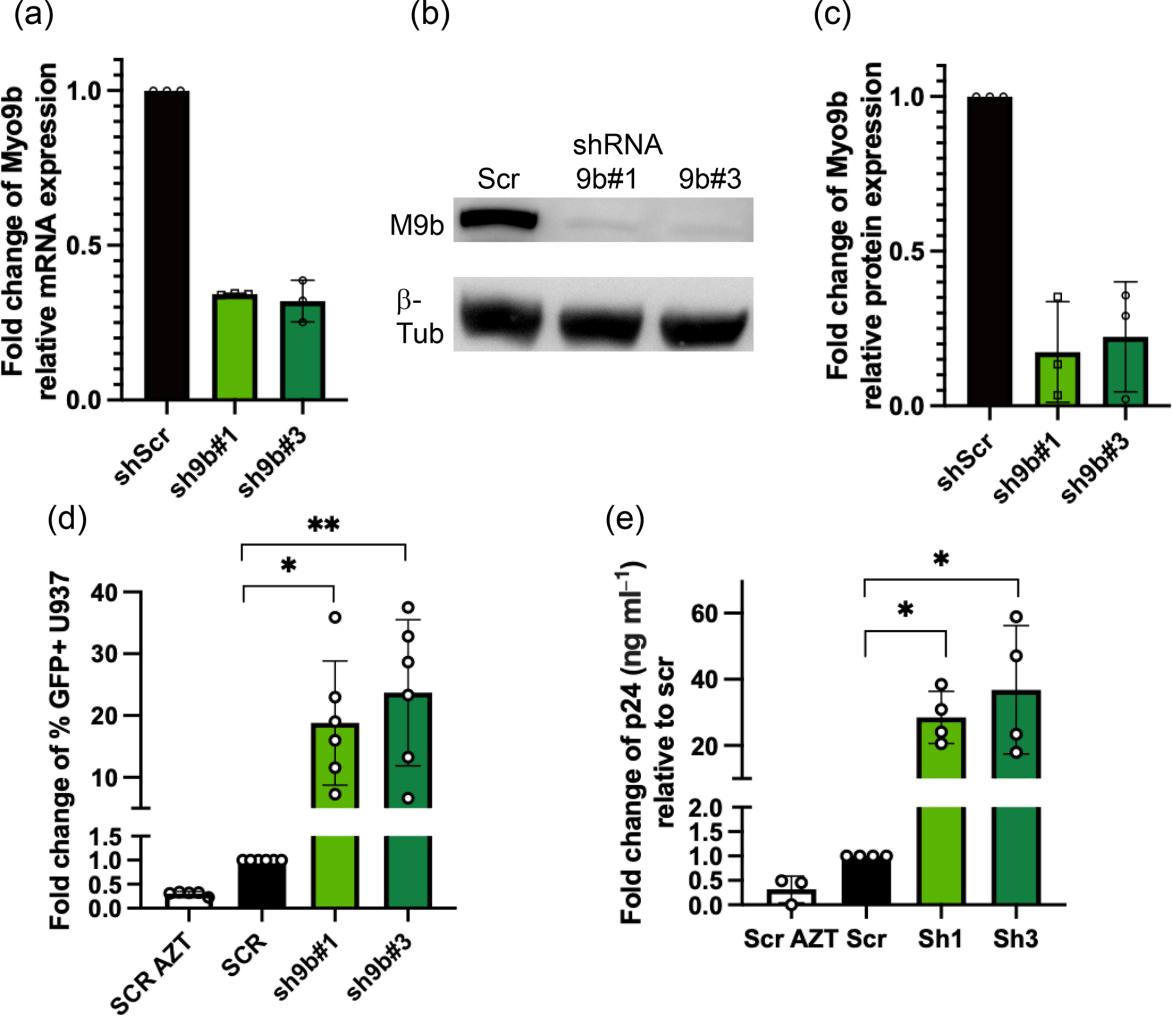
Myo9b knockdown increased infection of U937 cells with HIV NL4-3 IRES GFP VSV-G. Throughout the figure, U937 cells were transduced with lentivirus carrying shRNAs scrambled (Scr, negative control), Myo9b #1 or Myo9b #3. (a) Expression of Myo9b mRNA relative to B2M mRNA and normalized to shScr. The RE was calculated using the formula: 2^−(CtMyo9b−CtB2M). *N*=3. (b) Images of Myo9b and *β*-tubulin bands by WB. (c) Quantification of relative band intensity in WB. *N*=3. (d) Fold change relative to scramble of the percentage of GFP+ U937 cells infected with HIV NL4-3 IRES GFP VSV-G at MOI=0.05. Results from six independent experiments. *P<0.05, **P<0.01, Kruskal–Wallis test. (e) Fold change of p24 production in the supernatant of U937 cells infected as in (d). *N*=4. *P<0.05, Kruskal–Wallis test. Cells (d) and supernatant (e) were harvested at 48 h post-infection.

To test whether Myo9b had a role in HIV-VSV-G infection, we knocked it down in U937 cells using lentivirus-carried shRNA. We tested five shRNA sequences independently and chose to work with the two sequences that produced better knockdowns, i.e. an average of 80% knockdown at the protein level (Fig. 2a, b and c). We also chose to use, in our model, HIV-1 pseudotyped with VSV-G, as our clone of U937 cells expressed very low levels of CD4, CXCR4 or CCR5 (data not shown). Thus, the virus we have used behaves as a one-cycle virus. We infected control or Myo9b-silenced cells with HIV NL4-3 IRES-GFP VSV-G. By quantifying the percentage of GFP+ cells, we could ascertain whether the beginning of the HIV cycle (from virus fusion to integration, transcription and translation) was affected by the absence of Myo9b. By collecting and testing the supernatant of the infected cells for p24, we could detect alterations at the late part of the virus cycle as well as the beginning.

Interestingly, when Myo9b was silenced, we observed a strong increase of over tenfold in both the percentage of GFP+ cells (i.e. infected cells) and in p24 released to the supernatant (Fig. 2d, e). As the increase was seen in both variables tested, the step of infection affected was most likely at the beginning of the virus cycle.

Of note, when we stained HIV-GagiGFP-VSV-G-infected U937 cells for Myo9b and observed them at the confocal microscope, we found no co-localization between GagiGFP and Myo9b at 48 h post-infection (Fig. S1, available in the online Supplementary Material), suggesting that there is no direct interaction between Gag and Myo9b and that the increase in viral production could be due to the increase in infection. In addition, we found no differences in Gag localization between infected U937 cells expressing control levels of Myo9b (shScr) or cells silenced for this protein (shMyo9b #1 or #3) (Fig. S1).

### Myo9b knockdown also impacted the infection of Jurkat cells

**Fig. 3.**
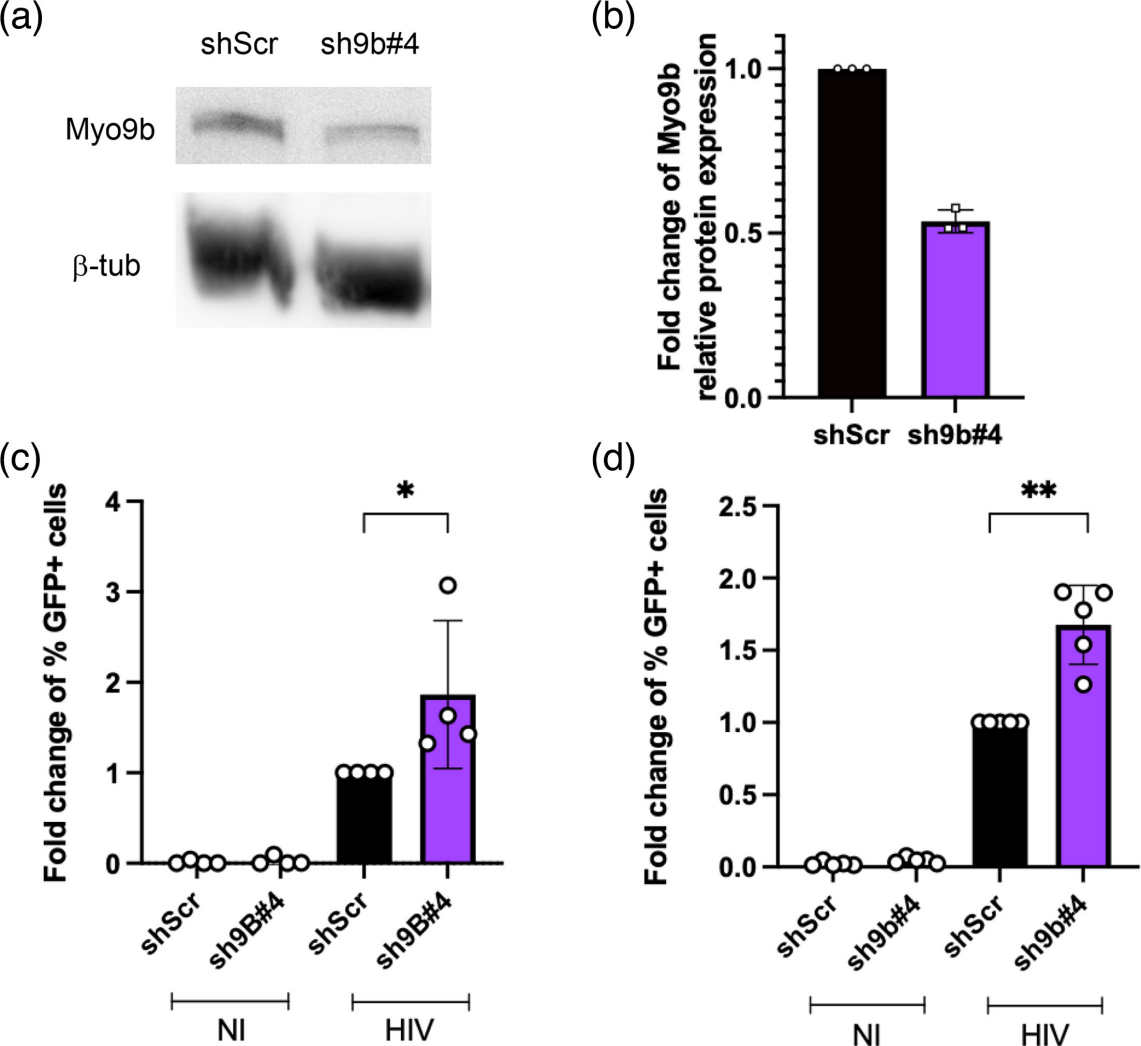
Myo9b knockdown increased infection of Jurkat T cells. Throughout the figure, Jurkat cells were transduced with shRNAs scrambled (Scr, negative control) or Myo9b #4. (a) Images of Myo9b and *β*-tubulin bands by WB. (b) Quantification of relative band intensity in WB. *N*=3. (c) Fold change relative to scramble of the percentage of GFP+ Jurkat cells infected with HIV NL4-3 deltaE EGFP VSV-G at MOI=0.1. Results from four independent experiments. *P<0.05, Mann–Whitney test. (d) Fold change relative to scramble of the percentage of GFP+ Jurkat cells infected with HIV NL4-3 IRES GFP at MOI=0.5. Results from five independent experiments. **P<0.01, Mann–Whitney test. NI, not infected.

To test whether the absence of Myo9b also affected the infection of a different cell type, we silenced Myo9b in Jurkat cells using lentivirus-carried shRNA (about 47% silencing, Fig. 3a, b). Jurkat cells were then infected with an HIV NL4-3 deltaE-EGFP-VSV-G virus, a virus that only enters cells via LDLR (as is the case of our U937 cells that express low levels of CD4). We observed an 86.4% increase in the percentage of infected Jurkat cells in the Myo9b-silenced population, as compared to control cells (Fig. 3c). Interestingly, the increase was not as expressive as seen in U937 cells.

We took the opportunity to test whether Myo9b silencing would affect infection with an HIV virus expressing only the HIV glycoprotein (gp120/gp41). HIV containing the NL4-3 Env glycoprotein can enter Jurkat cells using CD4 and CXCR4, by fusing directly at the plasma membrane, as well as by endocytosis. The preferred route is currently under debate [33,35]⁠. To check whether Myo9b could regulate the internalization of HIV carrying gp120/gp41, we infected Jurkat cells expressing shRNA Scr or sh Myo9b. As shown in Fig. 3(d), infection with NL4-3 IRES GFP HIV was also increased by 67% in Jurkat cells silenced for Myo9b as compared to control shScr cells.

### Myo9b-silenced U937 displayed an altered actin cytoskeleton

Myo9b contains a RhoGAP domain at its tail that can inhibit RhoA, B and C, and possibly Rac1 and Cdc42 [25,26]. RhoA has a role in inducing actin polymerization via mDia1. In addition, one of its main effectors, Rho-associated protein kinase (ROCK), activates LIN-11, Isl-1, MEC-3 kinase 1 (LIMK1), which inhibits cofilin by phosphorylating it. Likewise, Rac1 and Cdc42 activate the actin nucleation factor Arp2/3, as well as inhibit cofilin via P21 (RAC1) Activated Kinase 1 (PAK1)-mediated LIMK1 activation. Therefore, in the absence of the inhibition provided by Myo9b, we expected to find more p-cofilin and more polymerized actin in Myo9b-silenced U937 cells [13,17, 36]⁠.

**Fig. 4.**
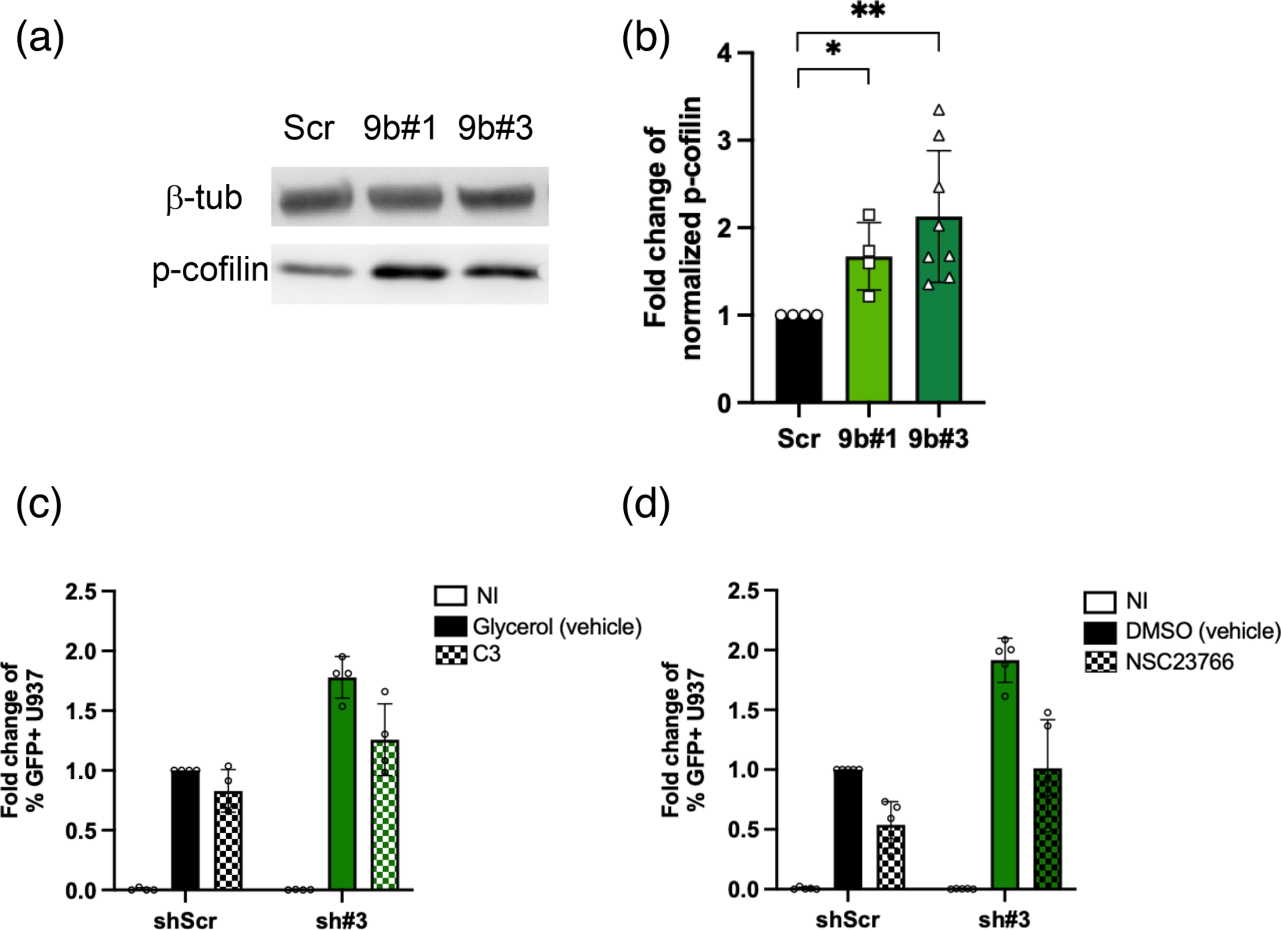
Cofilin phosphorylation in U937 cells expressing shRNA Scr or directed at Myo9b. (a) WB image for beta-tubulin and phospho-cofilin bands in one representative experiment. (b) The graph depicts the fold change of the levels of phospho-cofilin (normalized to either *β*-tubulin or GAPDH) in four independent experiments comparing both 9b#1 and 9b#3 to Scr, and four more experiments comparing only 9b#3 to Scr. *P<0.05, **P<0.01, Kruskal–Wallis test. (c, d) Inhibition of RhoGTPases reduces virus infection of U937 cells. Control (shScr) and Myo9b-silenced (sh#3) U937 cells were incubated with RhoA, B and C inhibitor C3 or Rac1 inhibitor NSC23766 for 4 h before infection with VSV-G-pseudotyped HIV-1 (MOI 0.01). *P*<0.01 (c) and *P*<0.001 (d), two-way ANOVA.

We then measured the amount of p-cofilin in U937 cells silenced or not for Myo9b. As shown in Fig. 4(a, b), we found more p-cofilin in cells silenced for Myo9b, as expected (mean of 67% increase for sh#1 and over 100% increase for sh#3). The increase in p-cofilin in Myo9b-silenced cells pointed to more active RhoGTPases.

To test whether inhibition of RhoGTPases could affect HIV infection in U937 control or Myo9b-silenced cells, we treated these U937 with inhibitors to RhoA, B and C (C3) or to Rac1 (NSC23766) for 4 h before infection with VSV-G-pseudotyped HIV-1. As shown in Fig. 4(c, d), both drugs reduced U937 infection by HIV-1. Such results suggest that higher activity of these RhoGTPases in Myo9b-silenced cells could be the reason for the higher levels of infection observed in Myo9b-silenced cells.

**Fig. 5.**
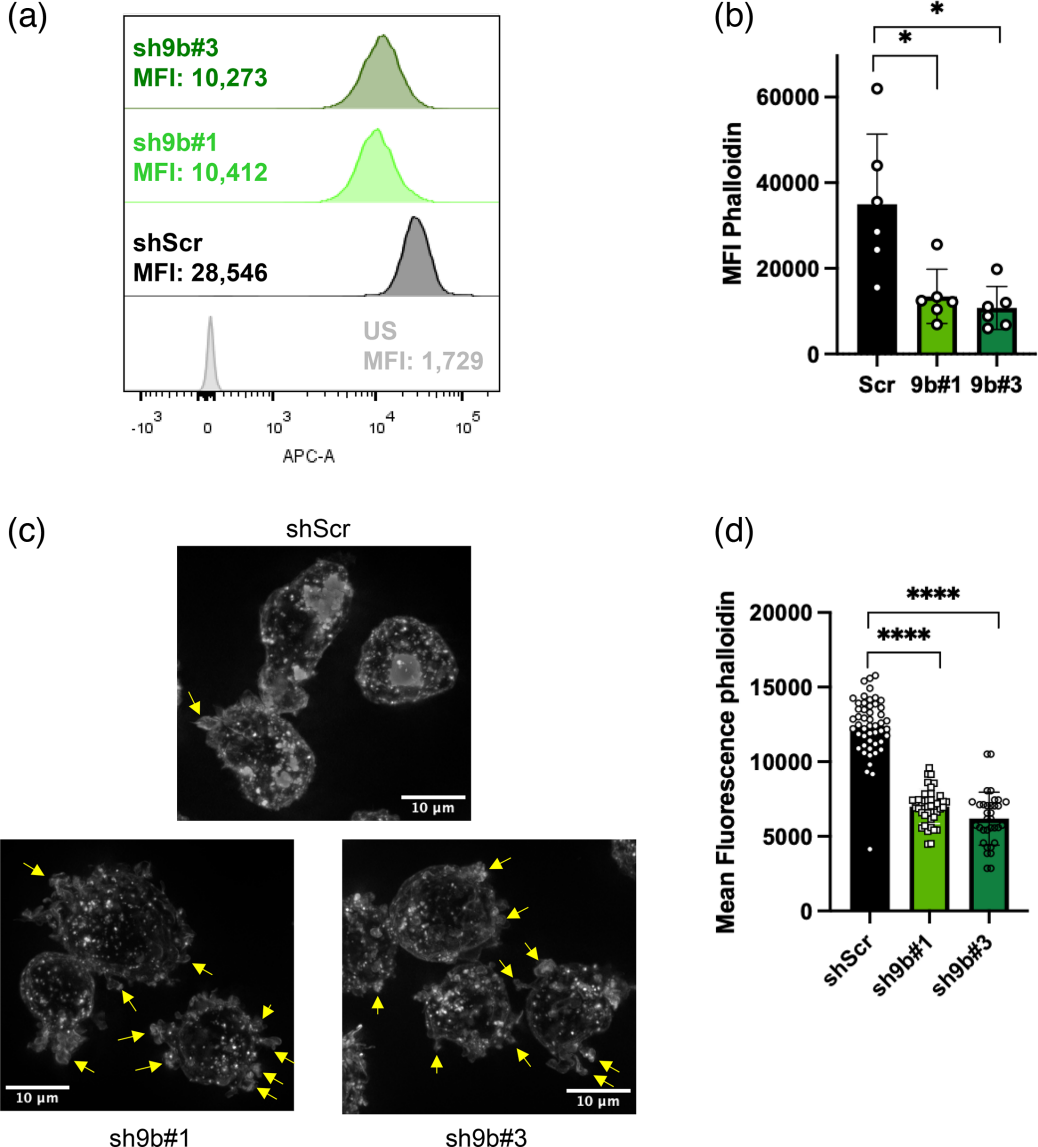
Polymerized actin content analysis in U937 cells. (a, b) U937 cells expressing shRNA Scr, Myo9b#1 or Myo9b#3 were incubated with phalloidin A647, fixed and analysed by flow cytometry. (a) displays an example of fluorescence intensity histograms obtained in one experiment. The light grey curve shows unstained cells, the dark grey curve shows shScr cells, the light green curve shows shMyo9b#1 cells and the dark green curve shows shMyo9b#3 cells. (b) shows median fluorescence intensities (MFI) of six independent experiments. *P<0.05, repeated measures one-way ANOVA. (c, d) U937 cells expressing shRNA Scr, Myo9b#1 or Myo9b#3 were adhered to poly-D-lysine-coated coverslips, fixed, permeabilized and incubated with phalloidin rhodamine. (c) Microscopy projections: images of different focal planes were obtained using a Leica DMi8 epifluorescent microscope and deconvoluted using LAS X Office software. Arrows point to examples of blebs. (d) shows mean fluorescence intensities for at least 30 cells per condition in one experiment. ****P<0.0001, Kruskal–Wallis test. This experiment was done once. US, unstained.

Next, we analysed polymerized actin content in control or Myo9b-silenced cells by staining them with fluorescent phalloidin. Cells were then analysed by flow cytometry and wide-field epifluorescence microscopy. As shown in Fig. 5, to our surprise, U937 cells silenced for Myo9b displayed a lower median fluorescence intensity of polymerized actin, as measured by flow cytometry (a mean decrease of 62% and 70% in cells expressing sh9b#1 and sh9b#3, respectively, Fig. 5a, b). The same effect could be visualized in microscopy images (Figs 5c, d and S2).

Image analysis also suggested that the knockdown cells had different morphology when compared to shScr control cells (Fig. 5c), showing many blebs, which could be compatible with a very active RhoA-ROCK pathway [37,38]⁠.

In conclusion, we observed more active RhoGTPases in Myo9b-silenced cells (as evidenced by increased p-cofilin) and lower levels of phalloidin-stained polymerized actin.

### My9b knockdown affects both fusion and internalization of HIV-VSV-G

VSV-G-pseudotyped HIV enters cells by LDLR-mediated endocytosis [39,40]⁠. With that in mind, we hypothesized that changes in the cell actin cytoskeleton could affect virus endocytosis.

**Fig. 6.**
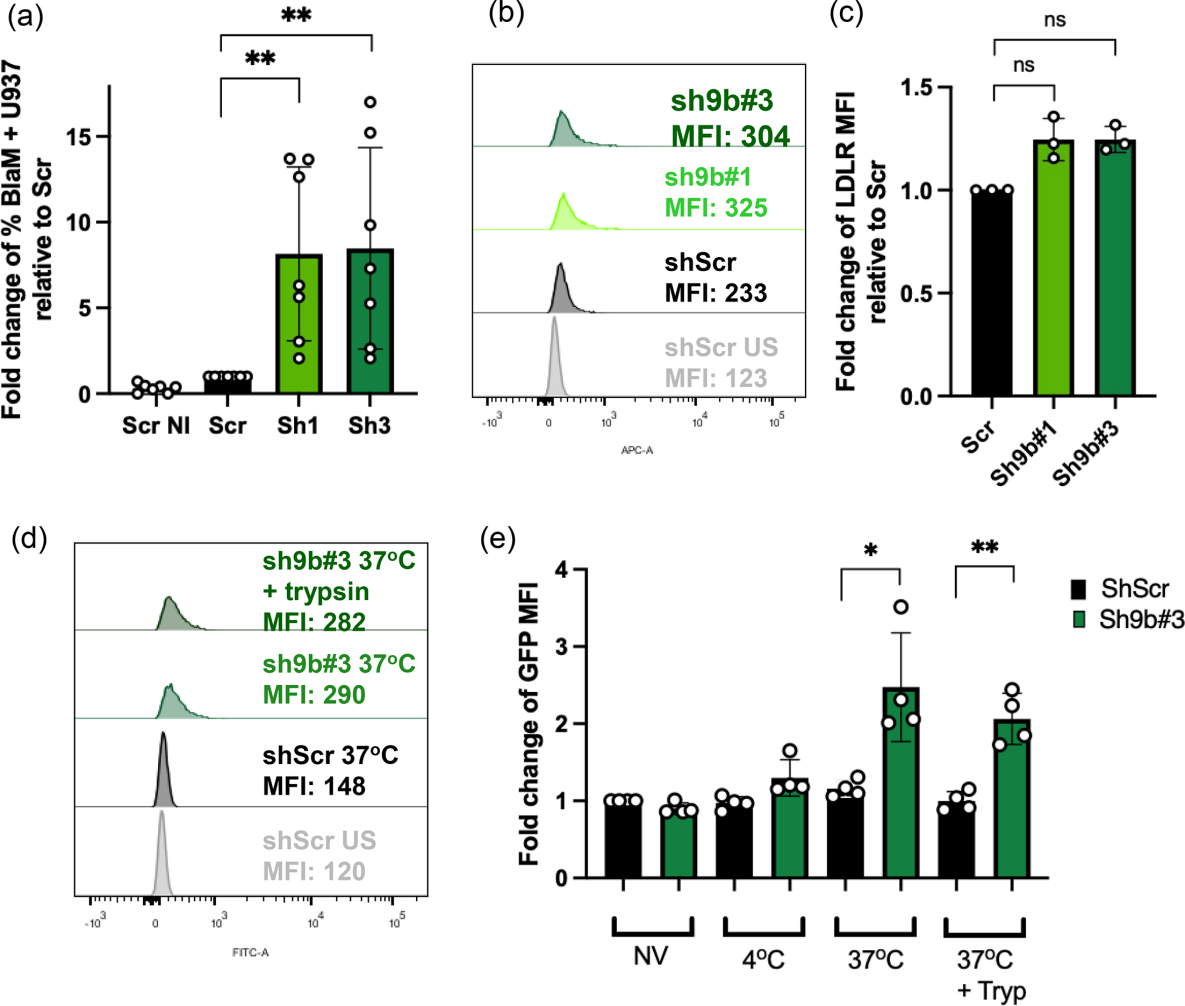
Silencing of Myo9b increases virus internalization and fusion. (a) U937 cells were infected using an HIV-BlaM-Vpr-VSV-G virus and loaded with *β*-lactamase substrate CCF4-AM. The graph shows the fold change over the Scr average of % BlaM+ cells (seven independent experiments using MOI 0.01–0.05). **P<0.01, Kruskal–Wallis test. (b, c) Cell surface expression of the LDLR in U937 cells expressing shScr or shMyo9b. (b) Histogram plots of one representative experiment. (c) The bars show the average fold change of the median fluorescence intensity of LDLR, from three independent experiments. Values were normalized to shScr. Differences are not significant, Kruskal–Wallis test. (d, e) Internalization by U937 cells (expressing shScr or shMyo9b#3) of VLP-GFP-VSV-G at the indicated temperature for 2 h, treated or not with trypsin before fixation, and analysed by flow cytometry. (d) Histogram plots of one representative experiment. (e) The graph shows fold change mean MFI in the GFP channel (of the total population) from four independent experiments. *P<0.05, ** *P*<0.01, Mann–Whitney test. NI, not infected. US, unstained. NV, no virus. Tryp, trypsin.

We first tested whether the membrane fusion step was affected by Myo9b knockdown. For that, we performed a fusion assay using an HIV NL4-3 BlaM-Vpr VSV-G virus, which allowed us to measure virus entry by the cleavage of fluorescent *β*-lactamase substrate. As shown in Fig. 6(a), the percentage of U937 cells to which viruses fused was higher among Myo9b-silenced cells. This experiment was repeated several times using different MOIs that varied from 0.01 to 0.05. As the infectivity of this virus varied significantly from batch to batch, the magnitude of the increase in fusion also varied. Nevertheless, compared to the shScr control cells, there was an increase in fusion with Myo9b-silenced cells in each and every experiment.

To evaluate whether the increase in virus fusion was due to an increase in the VSV-G receptor expression, we stained shScr and shMyo9b-expressing U937 cells for LDLR and analysed them by flow cytometry. As depicted in Fig. 6(b, c), although there seemed to be a slightly higher expression of LDLR on the surface of shMyo9b-expressing cells, the difference was not statistically significant, and we do not believe that such a small increase in receptor expression could account for the marked increment in infection.

To check whether virus internalization into the cells was also affected by the absence of Myo9b, we incubated HIV-Gag-GFP-VSV-G VLPs with U937 cells expressing shRNA Scr or shRNA directed at Myo9b. In addition, we treated part of the cells with trypsin after the 2 h of incubation, to detect only internalized particles and not VLPs attached to the cell surface. As shown in Fig. 6(d, e), after incubation with the equivalent of 2.5 µg of Gag in VLPs, we could not detect significant VLP internalization in shScr U937 cells. In contrast, shMyo9b#3-carrying U937 cells had detectable GFP fluorescence, even after trypsin treatment, indicating that reduction in Myo9b expression led to an important increase in virus internalization. These results suggest that the increased infection observed in Myo9b-silenced U937 could be due to an increase in virus internalization promoted by the derepression of RhoGTPases.

## Discussion

The actin cytoskeleton is a vital machinery in mammalian cells, and viruses infecting these cells need to establish a means of coping with actin. In some cases, viruses take advantage of the actin cytoskeleton to enter, be transported or exit cells (reviewed by [41]). In other cases, the actin cytoskeleton acts as a barrier to virus entry (as reviewed by [42]). Although much has been learned about the interactions between viruses and the cytoskeleton, much less is understood about the role of myosin motors in virus infection [4].

In this study, we have explored the role of Myo9b in virus infection using VSV-G-pseudotyped HIV as a model to infect a myeloid cell line (U937). We observed that Myo9b knockdown led to an important increase in virus infection and release. A similar, although less pronounced, increment was seen in the infection of the lymphocytic cell line Jurkat. We observed that Myo9b silencing also increased cofilin phosphorylation and reduced polymerized actin content in the cell. Finally, we observed that Myo9b silencing led to a rise in virus internalization and fusion, which could account for the higher levels of cell infection.

Myosin IXA (Myo9a) and IXB are unique among the myosin superfamily in that they possess, besides the typical actin-interaction and motor domains, a RhoGAP domain. RhoGAPs are RhoGTPase-regulating factors, as they activate the GTPase domain of the RhoGTPase, causing GTP to be hydrolyzed. Thus, the RhoGTPase remains attached to a GDP molecule, that is, inactive. The fact that Myo9a and Myo9b have this GAP domain makes them signalling molecules capable of self-transport. These myosins are probably recruited to discrete regions of the cell to exert their functions locally [5]⁠.

When Myo9b expression was silenced, the inhibition exerted by this protein on RhoGTPases was relieved. This was verified by the higher level of phospho-cofilin found in silenced cells, as was previously described [13,17, 36]⁠. As cofilin phosphorylation is carried out mainly by LIMK1/2 [43,44]⁠, which are downstream of RhoA, Cdc42 and Rac1 [45]⁠, it is difficult to ascertain which RhoGTPase was responsible for this action.

We also expected to find a higher content of polymerized actin in Myo9b-silenced cells, given the greater amount of phosphorylated and, therefore, inactive cofilin. In addition, RhoGTPases are known to induce actin polymerization through the activation of mDia1 (by RhoA), and Arp2/3 (by Cdc42 and Rac1) [46]⁠. However, we found consistently diminished staining for phalloidin in Myo9b-silenced U937 cells, as measured by flow cytometry and microscopy. When working with dendritic cells from Myo9b knockout (KO) mice, Xu *et al.* found a smaller accumulation of actin in immunological synapses [17]⁠. Although they did not quantify total actin, it is possible that the total polymerized actin content was indeed diminished. These authors mention that cofilin can act as an actin polymerizing factor by providing more filament pieces to nucleate, depending on the cofilin/actin ratio [17,47]⁠. On the other hand, it is tempting to propose that this decrease in actin polymerization might be due to a negative feedback loop in cells chronically lacking Myo9b, such as KO cells/mice and stable knockdowns (as we observed).

Although in mice and rats, Myo9b acts more specifically on RhoA [17,48]⁠, in humans Myo9b might regulate RhoA, Rac1 and Cdc42 [26]⁠. In addition, RhoGTPases are known to regulate each other, as RhoA inhibits Rac1, and also, Rac1 and Cdc42 inhibit RhoA activity [49,50]⁠. RhoGTPases are also regulated by dozens of other factors including RhoGAPs, RhoGEFs and RhoGDIs [51]⁠. Thus, the result of relieving Myo9b inhibition on RhoGTPases probably depends on the expression and activity of all these proteins and could vary according to the cell type. This could explain the less pronounced effect of Myo9b silencing in virus infection of Jurkat cells. Another explanation for that could be the knockdown level of Myo9b in Jurkats, which was inferior to what was observed in U937 cells (compare Figs2b  3b). Curiously, when we performed RhoGTPase inhibition in serum-free (in the case of RhoA, B and C inhibitor) or low serum (in the case of the Rac1 inhibitor) media, the difference in infection between shScr and shMyo9b #3-expressing U937 cells was reduced (Fig. 4c, d). It is known that FBS induces RhoGTPase activation [52,53], and therefore, the reduction in serum stimulation could lead to a decrease in the activity of RhoGTPases and, consequently, virus infection.

Interestingly, cell protrusions were also altered in Myo9b-silenced U937, as we observed several blebs in these cells. Bleb formation depends on RhoA-ROCK activity, as well as on myosin II (as reviewed in [54]⁠). Thus, a high number of blebs in Myo9b-silenced cells would be in line with a higher activity of RhoA.

Our results are in line with previous work showing that inhibition of RhoGTPases impairs HIV infection. For example, Watanabe *et al.* [55] have described that the super-expression of ARHGDIB, a RhoGDI and inhibitor of RhoGTPases, limits HIV-1 infection of the MT-4 lymphoid cell line. Lucera *et al.* [56] also found that RhoGTPase inhibitors restricted HIV-1 infection. del Real *et al.* [57]⁠ found that statins blocked HIV infection by impeding RhoA activation. Interestingly, Watanabe *et al*. have found reduced polymerized actin content by inhibiting RhoGTPases, whilst we have found the same by releasing RhoGTPase inhibition.

In our work, Myo9b silencing led to an increase in VLP internalization by U937, which could explain why a greater percentage of these cells were infected with VSV-G pseudotyped HIV. In line with our results, previous work has shown decreased internalization of the bacteria Shigella in HeLa cells over-expressing myr5, a Myo9b orthologue [25]⁠. In this paper, the authors concluded that, according to the pattern of protein recruitment by the invading bacteria, myr5 acted on RhoC, rather than RhoA. In addition, Quinn *et al.* [58]⁠ have observed significantly increased virus infection and internalization in cells over-expressing RhoC, but not RhoA, pointing to the possibility that the effects that we observed by silencing Myo9b could be due to augmented RhoC activity.

To summarize, our work confirmed that RhoGTPase activity is important for virus entry into cells and points to Myo9b as an important regulator of these molecular switches. Based on that, Myo9b emerges as a gatekeeper to protect immune cells from invasion by pathogens.

## supplementary material

10.1099/jgv.0.002090 Uncited Supplementary Material 1.

## Abbreviations

B2M: beta-2-microglobulin
GAPDH: glyceraldehyde-3-phosphate dehydrogenase
HIV-1: human immunodeficiency virus type 1
KO: knockout
LIMK1: LIM kinase 1
MFI: median fluorescence intensities
MOI: multiplicity of infection
Myo9a: Myosin IXA
Myo9b: Myosin IXB
p-cofilin: phosphorylated cofilin
PEI: polyethylenimine
qPCR: quantitative PCR
RE: relative expression
RhoGAP: RhoGTPase-activating protein
ROCK: Rho-associated protein kinase
shRNA: short hairpin RNA
VLP: viral-like particle
VSV-G: vesicular stomatitis virus glycoprotein
WB: Western blot
