# Supplementary material for: Myosin IXB protects immune cells from virus infection
Source: J Gen Virol. 2025 Apr 1;106(4):002090. doi: 10.1099/jgv.0.002090 (PMC11962068; doi:10.1099/jgv.0.002090)
Supplement: Uncited Supplementary Material 1. [file jgv-106-02090-s001.pdf]

A

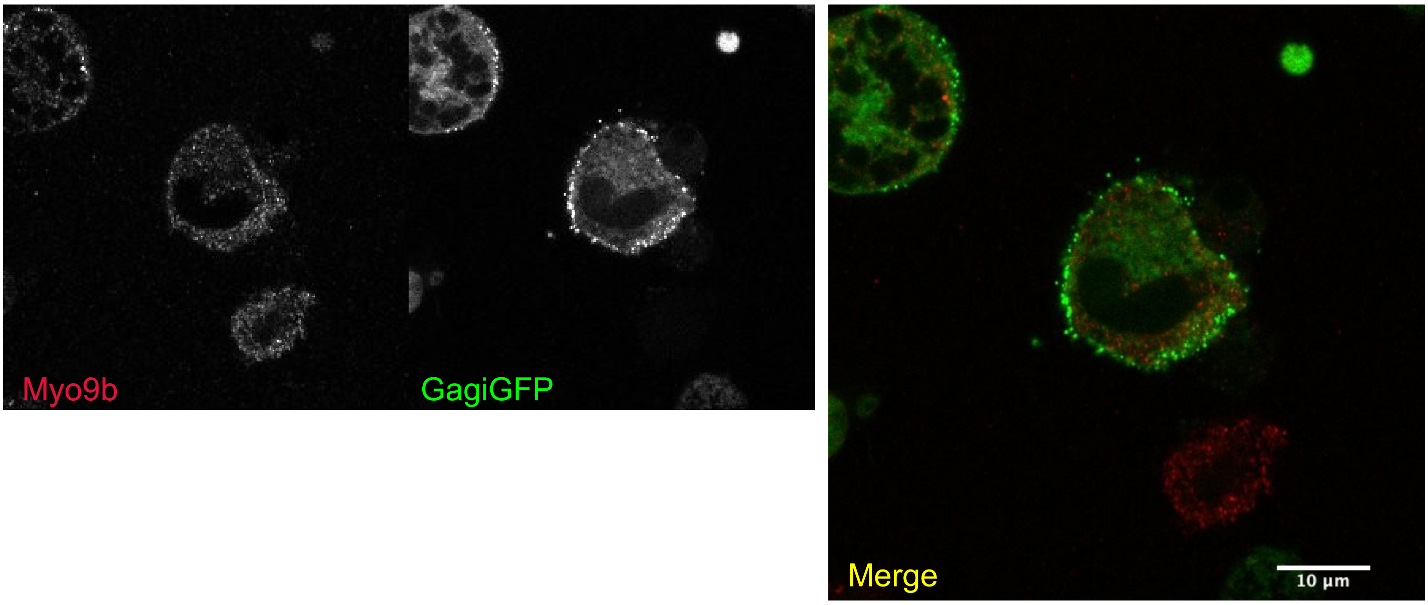

B

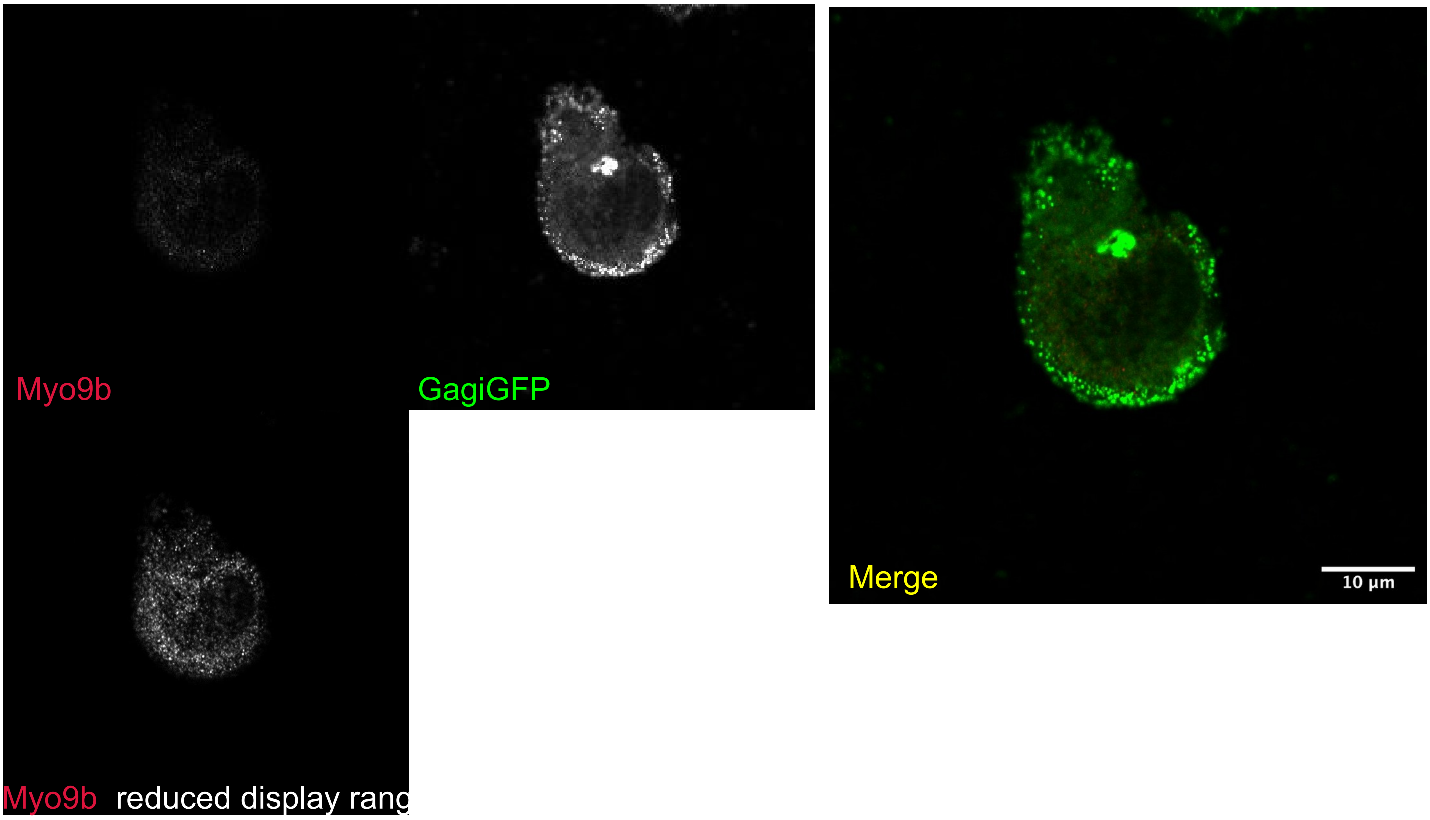

Figure S1: Gag and Myo9b do not co-localize in HIV-GagiGFPVSV-G-infected U937 cells. Infected U937 cells were adhered to poly-lysin-coated coverslips, stained with anti-Myo9b and anti-rabbit IgG conjugated to A658. Cells were analyzed in a Zeiss Axio Imager confocal microscope. A: U937 transduced with shRNA Scr. B: U937 cells transduced with shRNA Myo9b #3. Notice that Myo9b staining is hardly visible in this image (B) if the parameters are kept the same as for the Scr image in A. By reducing the maximum values for display range, then Myo9b staining can be detected. Images were obtained using an 100x objective. One focal plane is shown.

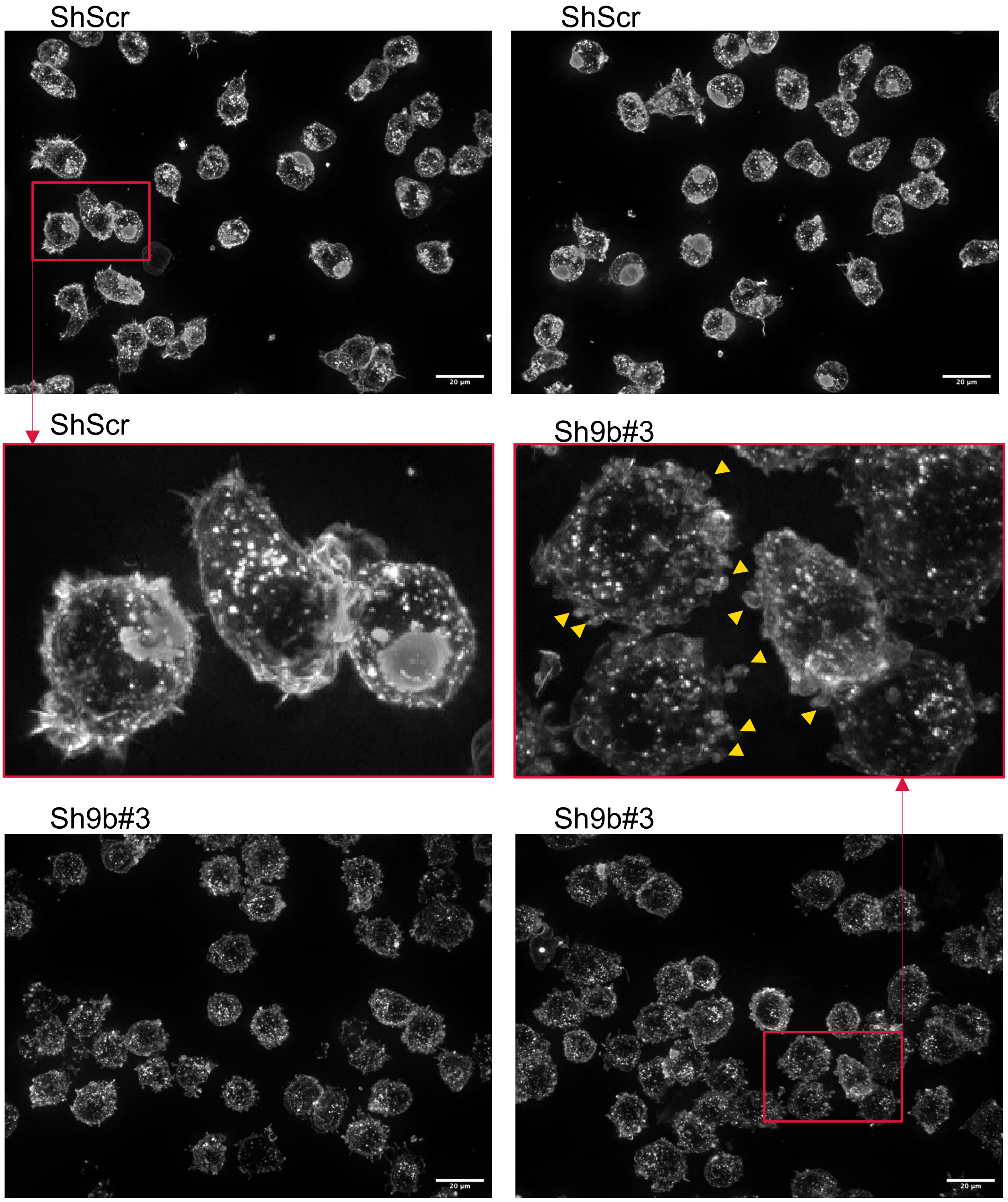

Figure S2: Phalloidin staining of U937 cells transduced with shScr or shMyo9b #3. Images were acquired in a Leica DMi8 fluorescence microscope at different Z positions and deconvoluted using a LAS X Office software. Scale bars at upper and lower images represent 20um. Middle images are magnifications of the inserts indicated.
